# “I felt like I was providing half a service”: Challenges, solutions, and action items for paramedicine when encountering patients experiencing intimate partner violence

**DOI:** 10.1371/journal.pone.0342542

**Published:** 2026-03-17

**Authors:** Rory A. Marshall, Tori N. Stranges, Nicole Merritt, Stephen Bartlett, Simon Sawyer, Paul van Donkelaar

**Affiliations:** 1 School of Health and Exercise Sciences, Faculty of Social Development, University of British Columbia Okanagan, Kelowna, British Columbia, Canada; 2 Physical Medicine and Rehabilitation, Michigan Medicine, University of Michigan, Ann Arbor, Michigan, United States of America; 3 British Columbia Emergency Health Services, Kelowna, British Columbia, Canada; 4 School of Clinical Sciences, Faculty of Health, Queensland University of Technology, Brisbane, Queensland, Australia; 5 School of Nursing, Paramedicine and Healthcare Sciences, Faculty of Science and Health, Charles Sturt University, Port Macquarie, New South Wales, Australia; Jordan University of Science and Technology, JORDAN

## Abstract

**Introduction:**

Intimate partner violence (IPV) is a pervasive and damaging global crisis. In response to the harmful health consequences, survivors often attempt to access the healthcare system. Paramedics are often the first point of contact with the healthcare system. Objective: Examine how the perspectives and experiences of paramedics may inform our understanding of current clinical practice and guide potential improvements for paramedicine.

**Methods:**

An interpretive description qualitative approach was used to design and conduct this research. Paramedics participated in focus groups discussing the intersection of paramedicine and IPV from the practitioner perspective. De-identified focus group transcripts underwent inductive pattern recognition. From the patterns, common challenges were identified. Corresponding solutions and action items were identified.

**Results:**

N = 17 paramedics (Women n = 7 (41%), Men n = 10 (59%); Mean Age 34 ± 10 years) participated in four focus groups. Even without clinical practice guidance for IPV, participants shared the service they were providing did not meet the needs of survivors. Common challenges at the intersection of paramedicine and IPV were: 1) patient barriers for help seeking, 2) individual paramedic disposition, 3) individual paramedic confidence, 4) paramedic service education, training, and readiness, 5) paramedic service guidance, 6) paramedic service configuration, and 7) interagency networks. Solutions and action items to address each challenge included updating functional education, training, infrastructure, and policy.

**Conclusion:**

Participants indicated that substantial challenges exist from the paramedic perspective at the intersection of paramedicine and IPV. Solutions and action items to bolster the education, training, infrastructure, policy, and positioning of paramedics were identified, providing positive direction. Meaningful, evidence-based implementation of these results should be pursued to advance the profession. Paramedics can be positioned as expert resources for survivors of IPV, linking through to vital supports that promote positive outcomes.

## Introduction

Intimate partner violence (IPV) is a public health pandemic. IPV is defined as the use of physical, sexual, psychological, and stalking behaviour by a current or former intimate partner to cause harm, and exert power and control [1]. This pervasive problem reportedly affects ~30% of women and ~25% of men in their lifetime [2,3]. Further, reporting methodologies, stigma, and other factors contribute to the under-reporting of IPV prevalence [2,4].

IPV is well documented to cause harmful sequela to the health and well-being of survivors [5,6]. An even greater prevalence of IPV is reported among individuals accessing the healthcare system [7,8]. IPV has been recognized as a clinical circumstance that is regularly attended to by paramedics. Paramedics (i.e., emergency medical technicians) are versatile healthcare professionals who primarily function in the emergency medicine and primary care domains [9].  Existing evidence suggests that survivors of IPV may frequently be attended to by paramedics, but IPV may not always be recognized by paramedics and survivors may not always be conveyed to the emergency department [10,11].

Paramedics are inherently interdisciplinary, spanning both in-community and formal healthcare settings. Recently, paramedics have been mobilized into other unique roles (e.g., community health, mental health, chronic disease management, etc.) as a point of contact with broad patient populations who have otherwise unmet healthcare needs [9,12,13]. As multifaceted professionals who can engage with both the healthcare and non-healthcare domains of IPV, paramedics could become well positioned to support this patient population.

To date, paramedics have been used in extremely limited capacities to support and care for survivors of IPV. In Canada, the Paramedic Association of Canada and the Canadian Organization of Paramedic Regulators provide scope and oversight for paramedic practice through the current National Occupational Competency Profile [14] and Pan-Canadian Essential Regulatory Requirements [15], respectively. Having multiple agencies providing oversight creates challenges to clarifying and introducing new scope. Neither Canadian governing body has specified that IPV is a clinical circumstance that paramedics should be educated, trained, and vocationally capable to encounter. Other important forms of violence, frequently termed abuse and neglect, are specified by both governing bodies when mapped together (e.g., violence against children, the elderly, or bariatric persons, etc. [14–16]), but IPV remains omitted. The general lack of direction given to paramedics and paramedic service organizations may underserve patients experiencing IPV. As IPV is well-documented to be cyclic and escalating, missing opportunities to intervene and appropriately care for patients experiencing IPV could leave survivors at risk of ongoing violence. Likewise, IPV can result in homicide, especially when survivors are attempting to leave a violent relationship [17].

A novel study conducted in Brazil highlighted the first characterization of IPV-specific patient care records within paramedic practice [18]. Importantly, this work provided clear evidence that paramedics are attending IPV calls, highlighted the first comprehensive characterization of patients attended to for IPV-caused reasons, and further highlighted documentation challenges occurring in ambulance patient care records [18]. In field documentation has previously been highlighted as a challenge in violence-related cases [19], indicating the need to use modalities beyond patient care records alone to assess the intersection of paramedicine and IPV. A survey from a sample of paramedics in western Canada examined their readiness to encounter patients experiencing IPV [20]. While paramedics were reported to have had strong knowledge of IPV, believed it was within their professional role to serve survivors, and showed a desire to help survivors of IPV, they felt less self-efficacy and emotional readiness when it came to the task [20]. This indicates a gap among paramedic readiness that is not related to education alone. These results aligned with findings from Australian paramedic students, showing deficiencies in readiness that may translate to clinical practice [21,22]. This means that potential gaps in clinical practice at the intersection of paramedicine and IPV may not be isolated to the Canadian context and global consideration may be warranted. Interviews with a sample of survivors of IPV who were attended to by paramedics for IPV-caused-caused reasons reported varied experiences, with common themes of poor interactions, lack of empathy, and insufficient support options creating barriers to care [11]. Further, focus groups with a sample of IPV advocates reported gaps in the care and services provided to survivors of IPV, with individual, service, and interagency level challenges faced by survivors [23]. While these initial investigations by paramedic services into the patient and advocate perspectives have been being explored, in-depth qualitative investigations into the paramedic perspective have still to be investigated. The combined input from patients, advocates, and paramedics would likely highlight areas for improvement holistically in these collaborative patient-focused interactions.

This study explored the intersection of paramedicine and IPV from the perspective of paramedics. It examined how the perspectives and experiences of paramedics may inform our understanding of current clinical practice and guide potential improvements for paramedicine.

## Methods

### Ethical approval

This study protocol was approved on March 1^st^, 2024, and then conducted in accordance with the permission of the University of British Columbia Okanagan Behavioural Research Ethics Board (#H22-02769). All participants provided written informed consent prior to enrollment in the study. This research was conducted in accordance with the World Medical Association Declaration of Helsinki.

### Approach and positionality

This research was conducted using a pragmatic worldview, relativist ontology, and constructivist epistemology [24,25]. Under this philosophical direction, an interpretive description (ID) approach was purposefully chosen to advance this work towards meaningful and functional outputs in the applied discipline of paramedicine [26]. As opposed to a uniform and strict research design protocol, ID provided a constructivist-rooted epistemological approach that could be molded to the specific needs and context of this research [26]. ID necessitates interpretation and description of data sources, in this study the transcripts and field notes from participant focus groups, in the contexts of the positionality of the researchers [26]. Thus, the experience and expertise of the research team are considered as valuable assets to be integrated in the co-creation of research between researchers (through interpretation and description of the data source) and participants (providers of the data source) [26]. This differs from many common qualitative approaches where the positionality and influence of researchers are considered bias that needs mitigating or removing, in favour of direct reporting of findings without contextual interpretation.

The research team was composed of clinician-researchers (RAM, NM, SB, SS) and researchers (TNS, PvD). These roles combined with the unique and diverse positionality of the research team were cumulatively leveraged to enhance the undertaking in alignment with the objectives of this work. The diverse clinical, research, and personal components of the research team members were mobilized in the research design to create and carry out a project that produced meaningful outputs in paramedicine.

### Participants

Self-identified English-speaking paramedics (18+ years) who practice clinically in the Canadian provinces of Alberta, British Columbia, or Saskatchewan were eligible to participate in this study. This region of Canada was selected as these three provinces share comparable paramedic education and training, paramedic scope of practice, population distribution, demographic make-up, and geographical proximity.

### Recruitment

Recruitment began May 1^st^, 2024 and concluded August 30^th^, 2024. Participants were recruited primarily via retained contact information from a survey study of paramedics [20] and through word of mouth. Gender balanced samples of paramedics from each eligible province were contacted in chronological order of survey completion to assess for interest in participation and provide more information. Willing participants were scheduled for focus group interviews, provided with the consent form, and advised to contact the research team with any questions, comments, or concerns prior to their focus group interview. No participants were excluded.

### Focus groups

Focus groups were consciously chosen over individual interviews to better replicate the team dynamic of paramedicine. As paramedics function in a team environment (i.e., most commonly responding to emergencies in teams of at least two), the research team decided focus groups would best display how each paramedic uses their voice and mindset when in groups of their peers, similarly as they may when practicing clinically. Focus groups have constraints (e.g., individual transparency limitations in a group setting, etc.), however this method was selected to better represent the group setting of paramedicine and how having others present may influence perspective sharing. All participants were made aware of the focus group setting and had the option to withdraw at any time if they did not wish to participate.

Given the general prevalence of IPV and the personal and vicarious trauma potentially experienced by participants, focus groups were conducted using a trauma- and violence-informed approach [27,28]. Principles of the trauma- and violence-informed approach (safety, trust, choice, collaboration, and equity) implemented into the design of this research include process design choices, facilitation practices, physical supports, ethical safeguards, and reflexivity [27,28]. All semi-structured focus groups were conducted using a flexible guide, built with safe strength-based and system-focused questions that avoided individual shortcomings, by a single trained and experienced researcher (RAM). Focus groups were approved to be conducted with between two and ten participants. The focus group facilitator had been trained to and was experienced in trauma- and violence-informed focus group facilitation, including managing overbearing inputs, creating space for all participants, and limiting the suppression of dissenting or minority views. The guide was made up of four primary areas: 1) current clinical practices in paramedicine in general and in situations involving IPV in particular, 2) challenges at the intersection of paramedicine and IPV, 3) solutions for paramedic-identified challenges, and 4) action items required for survivor and advocate-identified solutions. The survivor and advocate-identified solutions were gathered through previous research conducted by this team [23]. The findings from those projects were integrated into the experimental design of this research to facilitate timely utilization of the results in advancing the field. Paramedic-identified solutions from this study were also followed up with implementation needs. This approach was used to build actionable items from the best available evidence from patient, advocate, and paramedic perspectives. Participants had the option to participate in in-person, via video conference (University of British Columbia-licensed Zoom, San Jose, USA), or in a hybrid (in person and via Zoom) sessions with participant preference used to organize sessions. These three options were provided to participants to accommodate individual needs in the context of physical setting. Prior to each focus group interview, informed consent was obtained, demographic information to describe the participants was collected through a separate anonymous online survey, and established pre-focus group procedures were used to outline, discuss, and mitigate privacy and safety concerns (e.g., limitation of privacy within focus group, importance to not share discussed content outside of the focus group, etc.). Participants were encouraged to retain a copy of the consent form. All focus groups ran for approximately 60 minutes. All sessions were audio and video recorded, and field notes were collected.

All focus group interviews were conducted, to our knowledge, without incident. No privacy breach, safety concerns, or psychological upset were noted or reported during, immediately following, or during post-focus group follow up. Participants were offered $50.00 compensation for their participation. Two participants elected not to receive the compensation. Each session was reflected on by the facilitator (RAM) and other members of the research team in the context of trauma- and violence-informed research practices.

A combination of automated real time transcription (Zoom), line-by-line manual confirmation, and descriptive field note incorporation were integrated to engage with the data outside of standalone automated transcription [26]. All identifying and potentially identifying information was removed from transcripts during this process. De-identified transcripts were retained, and all original transcripts, audio, and video recordings were deleted.

### Analysis

Participants demographic items collected in the anonymous online survey were pooled and descriptive statistics were calculated (mean ± standard deviation or % (n/N) as appropriate).

Hybrid line-by-line open pattern sorting, combining principles of traditional open coding [29] and inductive data interpretation [26], was conducted to fully immerse the researcher (RAM) in the data by employing the rigor of traditional coding and the active interpretation of ID. The isolated use of the strict methods of traditional coding were avoided to allow the research design to be consistent with the objectives and applied functional outcomes. The cumulative data set was reviewed following each focus group (RAM). The interpretation of the data was reviewed by the research team through reflexivity sessions and revised if concerns of bias detracted from appropriate interpretation and description. Reflexivity sessions focused on identifying and avoiding application of pre-conceived notions and biases among the research team in the constraints of positionality. Data analysis (RAM) and collection (RAM) occurred simultaneously to continue design progression in the iterative process [26].

When the research team was satisfied with the hybrid open pattern sorting, patterns were evaluated, links were observed and assessed, and all open patterns were grouped into common patterns. Common patterns were housed under five categories 1) realities of paramedicine, 2) current practices at the intersection of paramedicine and IPV, 3) challenges at the intersection of paramedicine and IPV, 4) solutions for corresponding challenges, and 5) implementation needs for solutions. The realities of paramedicine was chosen as a category to provide context about the current state of paramedicine. The current practices at the intersection of paramedicine and IPV was chosen as a category to provide practitioner experiences of the existing clinical dynamics. Challenges was selected to understand what paramedics viewed as things to overcome when encountering patients experiencing IPV. The most impactful challenges were identified through group discussion of the findings among the research team. Solutions for the paramedic-identified challenges was chosen to provide functional guidance to overcome challenges and inform the field of paramedicine. Implementation needs was chosen to help action solutions derived by survivors, advocates [23], and paramedics. Implementation needs were separated into survivor- and advocate-identified solutions requiring implementation needs, and for paramedic-identified solutions requiring identification needs. Participants were asked directly about what they believed paramedics and paramedic services would need to enact suggested improvement solutions identified by survivors of IPV through interviews and IPV advocates through focus groups. The categories of solutions requiring implementation needs for survivor- and advocate-derived solutions for corresponding challenges were: 1) respect and believe survivors while using trauma- and violence-informed practice, 2) safe and appropriate direct questioning about IPV, 3) pathways to care and resources in situations of IPV, 4) referral, and 5) documentation into continuum of care [23].

As thematic saturation is typically used in ID methodologies [26], data collection ceased when paramedics from each available province had participated in the research, a reasonable effort to engage with potential participants had been undertaken, and timeline and funding constraints contributed to a natural conclusion. We acknowledge that more perspectives exist beyond what was gathered in this research. Common patterns were defined and presented with definitions and exemplary quotes as appropriate. Please note that overlap may exist between patterns as the complex and interlinking nature of this topic necessitates context across multiple points. As de-identified transcripts could not be and were not linked to demographic survey responses, exemplary quotes are not attributed to individual participants in this research. The term participants refers to a pattern or concept which was recognized or expressed by multiple participants. Instances where no dissenting views or disagreements were shared or indicated by any participants across all focus groups and instead views were shared or supported by all participants observed through words and actions (e.g., words of affirmation like “I agree” from transcripts, nodding agreement while saying “yes” reported in field notes, etc.) are represented as consistent across all participants. Given the small focus group size, observing all participants in each focus group was possible in real-time and further substantiated in data review. The focus group facilitator was conscious of and saw no indication of group think or suppression of dissenting views for any unanimous findings. While the methods used herein were not quantitative per idea and isolated views of individuals also provide relevant insight, the highlighting shared or unanimous sentiments was noted. This study aligns with and embraces the rigour of the Big Tent Criteria for Qualitative Quality [30].

## Results

### Demographics

N = 17 paramedics participated in four focus groups. A complete summary of anonymous self-reported participant demographics can be found in Table 1. The self-reported gender identifications (Women 41%, Men 59%) and age (mean 34 ± 10 years) of participants were comparable to the to those of the eligible population of paramedics (Women 37%, Men 63%, < 1% Non-Binary; mean 40 years [no standard deviation data available]) [20]. All other demographic information collected in this study were not publicly available for the eligible population. One focus group (n = 7) was conducted in-person and three (n = 3; n = 4; n = 3) were conducted virtually via video conferencing. No sessions were elected to be conducted using a hybrid (in-person and video conference) modality. Patterns were consistent across both elected modalities and modality type did not appear to affect the free flow of conversation in any of the focus groups.

**Table 1:** Self-reported demographics of participants who participated in focus groups about paramedicine and IPV.

Demographics		n	%
Age (mean ± standard deviation)		34 ± 10	
Gender	Men	10	59%
	Women	7	41%
Province	Alberta	4	24%
	British Columbia	7	41%
	Saskatchewan	6	35%
Cultural Background (multi-select)	European	17	100%
	Hispanic	1	6%
	Indigenous	1	6%
Race (multi-select)	Hispanic	1	6%
	White	17	100%
Marital Status	Married or Domestic Partnership	9	53%
	Single, Never Married	8	47%
Employment Status	Employed – Full-Time	17	100%
Income Status	Medium	15	88%
	High	2	12%

### Current realties in paramedicine

Participant experience patterns surrounding the current realities in paramedicine can be found in Table 2.

**Table 2:** Experience patterns and exemplary quotes learned from paramedics about the current realities of working in paramedicine.

Experience Pattern	Exemplary Quotes
Adverse Current Working Environment	“If you’re looking at a snapshot of has [the working environment of paramedicine] improved in the last couple of years? A little bit. And if you’re looking at as a profession, are we actually getting healthier and getting better? Absolutely not.” “People don’t want to come to work. And then when they do come to work, it’s like half a human is there half the time.”
Shifting Paramedic Service Utilization and Demand	“I think [emergency medical services] has been more and more filling the gap with mental health. And while we are a frontline stop gap for that. We certainly aren’t the solution.” “Since COVID people can’t cope. [The public] get a simple cold or flu-like symptoms and they think they’re gonna die. Then we have to take them in, and we’re tied up with these individuals for 8 to 12 hours on hallway waits. Even minor emerg. clinics are calling for very minor things that they can fix. Which pre-COVID would have been a no, but post-COVID if people are sick they go and see an emerg. doc which keeps pushing on the system. I think our resources are oozing. Like many of our urgent care clinics and medicare clinics have gone down too.” “You can see that it’s starting to weigh on, and every time the tones drop, and it’s coded as a high priority. read the notes [outlining the reason for the call], and they’re already pissed off going to the call.”
Under Prepared Paramedics to Meet Current Demands	“I do think that for the most part, people are drawn in for the acuity, right like, whether it’s you know, whether you prefer the blood and guts and trauma, or you want to really focus like your a big nerd for the heart stuff, and you like the, you know, the intricacies of some of these complex calls, like whatever it is. For the most part in my experience, that’s why people join, and so getting unfulfilled in that or feeling like you can’t do what you wanted to do because of these other calls. Next comes frustration and then that subsequent burnout, compassion fatigue, and that kind of path.”
Positive Progression Possible in the Future	“So, I think the mindset or kind of mentality has had a bit of a change in the last few years. A lot of that old mentality of the kind of “*Old Boys Club*” seems to be changed out, and it seems like in the environment they wanna support paramedics a lot more now.” “I liked the comment about the "*Old Boys Club*" going away. And yeah, that's definitely a massive shift in this organization. We’re not 100% perfect. There’s a lot of change to go still. But I can actually definitely see us trending in a good direction now.” “There’s a big push to fix [systemic culture issues]. And I do see a lot of new people that are really excited to be in the field.”

Many systemic issues were reported to be contributing to widespread staff burnout and reduced daily capacity. Excess middle management, being under resourced, being understaffed, being over worked, and being part of a largely ineffective healthcare system were highlighted. Participants explained that paramedic services were being stretched as a band-aid solution to address healthcare gaps. Given the increased demands, participants reported feeling underpaid for the updated scope of practice they were providing. Participants indicated there was a general state of discontent and low morale in paramedicine, further fueled by a lack of organizational support for paramedics. The sense of strained capacity to provide empathetic care was unanimous.

Participants cited a shift in paramedic service utilization and emergency department utilization. Participants explained that the patient profile was transitioning from strictly acute physiological health concerns to general health and well-being concerns with psychological, social, and environmental aspects. Complex patients across multiple spheres of needs were becoming the norm. From an organizational standpoint, minimal support had been given to participants for this change in utilization. While participants agreed that strictly high-acuity physiological emergencies (i.e., heart attacks, strokes, major traumas) were no longer expected, the public was calling 911 when it really was not warranted (e.g., “*stubbed toes,*” “*paper cuts,*” “*colds*”). In general, it was reported that among the patient population a lack of health literacy, lack of ability to cope, and diminished resilience were exacerbating the strain on paramedic services. Participants pointed at patient resistance and societal failures as contributing factors. Participants reported more calls were being dispatched as high acuity (i.e., warranting a lights and sirens response), when in fact the reality of the call was low acuity. This was reported to increase stress from being forced to drive under riskier circumstances on a chronic basis. Participants reported that mental health emergencies had become increasingly common, which they felt ill equipped, under trained, and under resourced to manage. Toxic drug poisonings (i.e., overdoses) were also reported to be an ongoing challenge that occurred multiple times over the course of most shifts. Participants indicated that this trending shift had grown exponentially post-pandemic. The aging population was also reported to be a factor in the perceived increased call volume.

Considering the adverse environment of paramedicine and the change in demand, participants shared feeling generally under trained and not prepared to manage the things that were being asked of them. From an organizational standpoint, minimal support had been given to participants for this change in utilization. Reoccurring problems bothered participants because they felt they had lost the ability to actually help. All participants reported they wanted to meaningfully help address all patients’ health concerns.

The make-up of paramedic services was reported to be shifting positively away from the “*Old Boys Club*” mentality (a closed informal network of people who support each other limiting the progression of others) and staff model towards a more junior but better educated staff with a progressive and informed mentality. Paramedics with outside sources of education (e.g., degrees from other fields, etc.) were reported to be entering paramedicine. Participants reported there was an appetite for more education, training, and options to manage the demands of contemporary paramedicine. The dichotomy between general discontent, and the progressive mindset and professional shift was noted by participants as being an ongoing complexity. Participants indicated there was hope for the advancement of the profession but were concerned that if the overall state of paramedicine did not improve, then there would be a mass exodus from the field. Participants were aware of and described the current environment working in paramedicine as challenging, complex, and dichotomous.

### Current realities in paramedicine and IPV

Participant experience patterns surrounding the current realities in paramedicine at the intersection with IPV can be found in Table 3.

**Table 3:** Experience patterns and exemplary quotes learned from paramedics about the current realities of working in paramedicine at the intersection with intimate partner violence (IPV).

Experience Pattern	Exemplary Quotes
Circumstances when Attending IPV Calls	*“*[The call] was known for intimate partner violence, and it’s a wait for the police, and then you show up and the police are already there, and everything’s diffused. And people are mad, and they don’t talk to the police, so they don’t want to talk to you. And so, you try your best, and [the people involved in the incident] don’t talk to you. So, you hop in the ambulance, and you leave.”
Uneasiness when Attending IPV Calls Despite the Desire to Help	“It’s a very awkward conversation to be having, be it domestic assaults, sexual assaults, whatever. I don’t think that we’re properly trained in handling that. I imagine that's, ya know, because everybody just wants to tiptoe around it.” “You start to advocate for them. So, it’s up to the practitioners to learn how to how to go about that. And generally, that’s done by just by recommending transport to hospital to speak to somebody who can help them. Which is vague and tricky, and not overly convincing for patients.” “It’s not a really clearly defined role other than the black and white, make sure they don’t have a life-threatening injury and advocate transport. Our scope could easily be broadened.”
Ambiguity and Lack of Direction	“I think as the attendant…trying to bring that person to services for their needs [is important], but sometimes it’s hard to do inside the algorithm and I don’t think we even have an algorithm for this.” “I feel like a spectator. Either you show up and something doesn’t sit right and you’ve kind of got that inkling in the back of your mind. So, you know, we’ve got very limited training on it but you do what you can.” “In my experience, I felt like I was providing half a service. I’m there and I’m basically just there to check you out and make sure that you’re not dying. But then, that’s not what they’re interested in right now. You know, and I’m not gonna try to offer a lecture to someone who needs a hug. But I’m just there. I do a physical exam, and then I shrug my shoulders, and I look at the cop, and I say “*now what?* *Are they pressing charges?*" I have no idea where to go on these things. If I don’t have someone there, like the police kind of pushing things along, then I don’t know what to do next.”

All participants reported they had attended calls for IPV, and most had attended one or more calls for IPV homicide. Ambulance calls for IPV were reported to be frequently initiated by police request, co-responded to with police from initial dispatch, or were dispatched as something different all together. Participants indicated that when co-responding with police, the tone of the interaction was set by the attending police officers. Participants recognized and appreciated the role police played in safety for both the paramedics and the other parties involved, but did see it as an inherent limitation.

Participants explained that calls for IPV often felt uncomfortable and awkward. There was a strong desire to help survivors of IPV, but participants explained they had little to offer. Participants who identified as women shared they thought they had better success at building rapport and trust with patients than participants who identified as men, especially when responding to calls where the perpetrator was a man. Creating a safe space, treating people with respect, separating the patient (survivor of IPV) and the partner (perpetrator of IPV), and ensuring private interactions were reported as common techniques to manage scenes. Participants explained transport to the emergency department was not appropriate, except in cases where patients had injuries requiring treatment, because the patient’s needs would not be met in the emergency department. However, participants reported that transport to the hospital or leaving patients in-community were their only options, and that transport to the emergency department may result in ongoing care where as non-conveyance would not. Participants reported imposing their personal beliefs onto patient situations and being frustrated in repeat attendances.

Ambiguity was a constant across interactions, protocols, and procedures. Participants reported there was a lack of options and process for calls involving IPV. There were concerns around balancing privacy and questions to accurately interpret the situation. Participants reported not knowing what or how to ask about IPV. Moral injury was identified as an area of concern as participants shared they could not support patients while practicing within their given directives. For example, participants reported having their scope limited to transporting patients to approved healthcare facilities or having patients refuse transport to approved healthcare facilities. As participants reported shelters were not approved healthcare facilities, referring patients to these facilities could put a paramedic’s license or job at risk. Participants frequently used non-sanctioned work arounds to try to meet patient needs as best they could (e.g., calling shelters on behalf of patients, providing transportation to shelters), and acknowledged this was putting themselves in a compromising situation if anything went wrong. This practice was also practitioner dependent. Participants reported challenges around reporting, not knowing if they had to or how they were to report IPV. Further, documentation was a concern as participants reported trying to document objective facts but explained feeling limited when there was no disclosure of violence, or they had suspicions without direct confirmation. Participants reported their documentation would be extremely varied. Outside of their physiological findings in their patient care records, a verbal handover of the violence suspicion or safety concern would be given to emergency department staff if the patient was transported. Participants reported that some patients would ask them not to document IPV. Participants reported slight knowledge of specialty teams from other agencies but conceded that there were minimal or absent processes for collaboration.

The current realities of paramedicine and IPV were cumulatively reported to be responder dependent, inconsistent, unstructured, ill informed, and failing to meet the needs of patients.

### Challenges in paramedicine and IPV

Many challenges were identified by participants. The seven most impactful challenges (identified by the research team based on participant input) and exemplary quotes can be found in Table 4.

**Table 4:** The seven most impactful challenges reported by paramedics when encountering patients experiencing intimate partner violence (IPV) and exemplary quotes.

Challenge	Exemplary Quotes
Patient Barriers to Help Seeking	“They’ll say, “*I just got my face kicked in by my husband. I don’t want to talk at all.*” “There’s been a few instances where I’ve really wanted to take the person in to the hospital and they just won’t come. And then you’ve got to sit with that.” “I’m assuming [the patient] just gets left at home, and then call us back in 2 days, when it happens again.” “I can’t count the amount of times I’ve heard, “*he’s nice when he’s not drunk. Our relationship is great when we’re not drinking together.*” And a lot of times that’s when they get passed off by police or by other services while they sober up and the window gets missed.” “Lots of [IPV] calls also involve drugs and alcohol. And so, a patient who maybe is slow to respond, or appearing dazed can easily be written off as behaviorally non-compliant or a little intoxicated, which isn’t fair to them. Admittedly often is the case but doesn’t negate the fact that it’s not always that.” “I’ve had a few [IPV] calls involving strangulation and the patient, or the partner doesn’t want to go to the hospital. And for me, it’s a hard line to walk, right? Because the aftereffects of strangulation. It can come so much further down the line.”
Individual Paramedic Disposition	“These aren’t [ABCs; Airway Breathing Circulation; foundational basic physiological essentials in emergency medicine] and [ACLS; Advanced Cardiac Life Support; premier advanced resuscitation program for cardiac arrest]. These are like real human situations, and that’s where the burnout shows even more than our pre-programmed stuff that we know how to do in our sleep.” “I think, I will very typically put my own ideals of what family life and relationships and whatever else should look like onto people. And then when what they’re experiencing doesn’t match, like, okay, well that’s wrong.” “When now you’re monitoring, say your partner as well as a patient, they can just create an environment that doesn’t exactly support paramedics.” “You don’t even get the lip service. It’s just, “*yeah, everything I’m seeing here checks out. Do you want to come to the hospital? Yes, or no?*””
Individual Paramedic Confidence	“There’s no follow through. It’s an “*oh, he hit you? Shoot. What a problem”,* right? We have no solution that we can provide.” “I think it’s a little bit hard to find the line of making sure that we’re getting the best patient care possible, but also keeping that patient safe once they’re out of our care, right? You know, we can fix them on scene, but you know what we leave them with back home can be a whole lot worse.” “I think part of it could also be just be related to general hiring practices of [emergency medical services]. I feel like right now we’re dying for staff, as mentioned earlier. So, there's this desire to fill bums in seats. I think that some of our staff might not always have the same motivations and intentions that other practitioners do have.”
Paramedic Service Education, Training, and Readiness	“I don’t have a clear understanding of what I’m trying to refer [IPV patients] to. So, when I’m trying to refer them to some generic victim services, or to the hospital to talk to somebody. I’m not fully understanding what their process is going to look like. So, it’s harder for me to prepare them for it and to pitch it to them.” “I don’t think there is a lot that is given to us a training tool, a soft skills capacity and understanding what’s going on. I know in school at both the primary care and advance care paramedic levels that it’s hardly touched on.” “We all know how those systemic flows go. We don’t have anything devised in a way that can help us navigate the emotional side of things, like the mental side of anything.” “I think like what everybody was saying before we don’t have the tools to go down that road and I think we’re uncomfortable, so then it just makes it seem disjointed because you’re like “*ahhh, I don’t know if I should ask that but that seems like legitimate question to ask*”, and when most people are withdrawn or whatever it, like, we don’t want to go and bother them.”
Paramedic Service Guidance	“Intimate partner violence frequently is a recurrent pattern. It’s usually not a one-time event, so, I know that when I’ve offered contact to police for that, it’s been met with resistance because “*I’ve called them before, and all they did was say, thank you for telling us*,” and moved on. And there’s no true resolution that occurs in that situation.” “You start to advocate for [the patients]. So, it’s up to the practitioners to learn how to how to go about that. And generally, that’s done by just by recommending transport to hospital to speak to somebody who can help them. Which is vague and tricky, and not overly convincing for patients.” “There’s certain things that we don’t need to ask. We all know to look at their neck, to look, listen, feel their chest, but on those calls, we don’t have the tools to go down that road and address the emotional side of things.”
Paramedic Service Configuration	“So, even if a [victim services phone] number is available, you get put on hold for half an hour or 45 minutes while you’re trying to process something. The probability of staying on the phone decreases as those minutes progress. So, from being able to provide limited access to patients, it’s not even necessarily helpful.” “Our social workers don’t work at nighttime either. So sometimes you’ll go in and it'll be like 2 in the morning. You bring your patient in. Ask if they’ve got social work available. They’ll be like "*they’re not on until seven or eight* o'clock". So, you’re like, well, what good is this? They’re gonna sit here for, you know, six hours to see a social worker that comes in the morning. It seems counterproductive.” “To tie back to kind of like what I’ve seen about the division between the legal and the medical side of things. Then I find myself. I’m writing my narrative, and do I include the part where they say, you know, “*sometimes he punches my head*” like, you know, things that will help an investigation so when I get subpoenaed, my [patient care record] is useful to the victim in court, or am I here like giving a medical report? Am I here, covering my own behind with my [patient care record] to say I did what I was supposed to do? It’s a very strange process. What I include and what I don’t include is what I’m comfortable with.”
Interagency Networks	“Well, every single piece needs to fit together. Every single, person that talks or is around that patient has to be kind of on the same page. You know, if there’s 10 of us on scene, and nine of us are doing our damnedest and then that 10th guy just says something offside, or he says something snarky, or whatever the whole thing unravels. It just takes one comment, and then the whole thing is gone.” “In my experience it’s however interested whatever nurse I’m handing care off to is, and that’s about it. I get a triage nurse who’s sometimes not interested in what’s going on and then, you know, we’re going to a low acuity side of emerg. So, it’s usually like a streaming waiting room or something like that. You know, I’m not gonna give a report to a bedside nurse, not gonna talk to a physician. They’re churning through a ton of people…So, it’s not their fault, but it’s still it’s brutal. It’s absolutely just lost. These patients are just lost in the machine.” “There’s not a lot available in our resources that can be given out or easily found, things are expensive, or it doesn’t come with the financial support and especially the post-COVID world.” “We’ll drive to the hospital. They’re non-urgent, they’re not acute, usually, so, they don’t get seen right away. So, then they get moved to the waiting room, and they get fed up with being in the waiting room because it’s probably ideally not the place for them to be surrounded by the things that are usually in the waiting room. And then they leave. And then we just rinse and repeat and then something can swap and happens where they didn’t get help and something went too far or whatever, so, it’s like I don’t know what the fix is.” “I should say that there’s a lack of resources available.”

Participants reported that patient barriers to help seeking (the willingness of patients to engage with paramedics) was a consistent challenge on calls involving IPV. Participants shared “approachability” was affected by personal and situational reasons (e.g., acceptance of violence, love for the perpetrator, stigma, refusal of support, worsening violence following service use, safety concerns, perpetrator dependence, etc.), and service provision reasons (e.g., no faith in paramedic services as resource, previous negative experiences, etc.). Participants reported that patients having to re-tell their story repeatedly presented a barrier to help seeking as survivors were observed to feel that they were not being heard, and subsequently turned off from seeking help. In situations where substance use was a factor, participants reported patients were perceived to be more closed off because they were likely to have been mistreated by paramedics for substance use complaints in the past. The presence of police and corresponding level of aggression used by police were also speculated to impact patient help seeking behaviour.

The disposition of each individual paramedic (the demeanor and mental standpoint of each paramedic to deliver empathetic and appropriate care) was theorized to be affected by many factors. Workplace conditions (e.g., “*call volumes,*” “*burn out,*” “*jadedness,*” “*poor dispatching*”) were believed to impact each paramedic’s disposition. Negatively perceived workplace conditions were speculated to exacerbate harmful personal beliefs (e.g., *“racism,*” *“cultural discrimination,*” “*uninformed attitudes*”). Participants reported that while some paramedics were great to work with at all times, harmful deep-rooted personal beliefs of others were often present. A negative disposition was recognized to impact how approachable the patient was or became, as well as the entire call. Participant gender and on scene safety were also factors that affected disposition.

The confidence of the paramedic (the belief within the individual paramedic that they can provide appropriate care, and that appropriate care options are available), divided into self-efficacy and systemic efficacy, was identified to create challenges for service provision. Participants reported not feeling confident in knowing how to act, what to say, or how to say it. These feelings were worsened as participants reported not knowing how or where to progress the call to. In addition to the lack of self-efficacy, participants had no faith in the system. Participants believed there were not the means in place to appropriately support survivors of IPV. Thus, participants reported they were even more hesitant to act during calls involving IPV. Knowing they did not have the ability to meaningfully support survivors of IPV further impacted the self-efficacy of participants. The evolution of hiring practices within in paramedicine was reported to reduce the trust participants had in certain paramedic partners, synergistically contributing with self-efficacy and systemic efficacy to personal confidence concerns in situations encountering IPV.

Overwhelmingly, participants explained they lacked the knowledge, skills, and general readiness to manage situations involving IPV (the functional information, practical skills or behaviours, and readiness that paramedic services situate staff to possess and utilize). A lack of IPV-specific training, a lack of de-escalation training, a lack of screening questions, and an overall lack of preparation to manage social and mental health calls were indicated to negatively impact the training and readiness position of participants by paramedic services. Participants also shared feeling ill-equipped to consider IPV-caused brain injury and strangulation (which all participants reported having encountered). Participants reported not looking to deliver counselling or specialist services but rather indicated they felt under-trained and not ready to support patients implicitly at even a basic level.

Participants reported a complete lack of guidance (the recommendations paramedic services create and deliver to direct staff how to manage a situation) from paramedic services about how to manage cases of IPV. All participants reported they did not have or were not aware of clinical guidance on care practices for cases involving IPV. A general lack of process and lack of referral options were identified as guidance deficiencies. Further challenges were reported around documentation. With no uniformity or standards being outlined, participants indicated they were unsure of what and how to document and for whom they were writing the documentation. Participants also expressed concerns of liability depending on their documentation. Participants indicated they were doing what they thought was best but had no comparator. Overall, participants explained there was no IPV-specific clinical practice guidance and that this impacts their ability to navigate these situations in a multifactorial manner (e.g., standard of care, legal obligations, self-efficacy to serve, etc.).

Mirrored by the lack of guidance, participants explained there was no systemic infrastructure (the infrastructure paramedic services create and maintain to facilitate staff adhering to the advised guidance to manage situations) to support any practices around caring for survivors of IPV. Participants agreed that the emergency department, in its current capacity, was frequently the wrong destination to meet the needs of survivors of IPV. Further, participants shared the lack of configuration of systemic alignment had large gaps including a lack of pathways, a lack of processes, a lack of continuum of care, a lack of referral options, a lack of transport destinations, a lack of non-conveyance options, and being generally under resourced. Participants shared there was systems level organizational ineptness that left no practical options for paramedics to meaningfully care for survivors of IPV. Participants shared their frustrations that even when they had made gray area decisions to best accommodate patient needs, there was no return communication on if their decisions were effective or useful. The lack of infrastructure for IPV cases was also in the general setting of under resourced and understaffed paramedic services. Participants explained there was no infrastructure in place to support consistent progression of calls involving IPV.

Participants reported that the process and general network among services that support survivors of IPV (the composition, organization, and implementation of available service providers that may be concurrently or sequentially deployed to support in people in certain circumstances) was disjointed and deficient. Participants said that while paramedic services could perform some vital functions to support survivors of IPV, that there were expert resources who could be more holistically used to support the needs of survivors of IPV. Challenges reported by participants about this process, or lack thereof, included a lack of interagency progression of service delivery, a lack of defined process, a lack of referral options, no continuum of care between services, and not having the services in accessible places (e.g., limited resources in the emergency department, limited mobile services). Care for children which did not involve child services was noted as a major deficit. Survivors of IPV were reported to refuse transport to instead care for their children in favour of having child services involvement and potentially losing custody of their children. Participants shared that while many expert services did exist, the discombobulated nature of paramedicine within the context of these services left participants within no practical options to meet survivors needs. This left survivors of IPV at high risk of having a poor experience which did not meet their needs, a challenge which participants felt would lead to reduced service access attempts. Participants noted there were many systemic issues with services and resources for survivors of IPV, but those were beyond the scope of this work.

### Implementation needs for survivor and advocate-derived solutions for paramedic services

Survivor and advocate-derived suggested solutions from previous research [23] to improve the intersection of paramedicine and IPV, paramedic-identified implementation needs for these solutions, and exemplary quotes can be found in Table 5. All five suggested solutions were also identified by the paramedic participants herein in response to challenges identified by the paramedic participants, independent of the *a priori* specified line of questioning dedicated to address these issues in the semi-structured interview guide. Co-identification of the survivor and advocate-derived solutions, and paramedic-identified solutions indicate alignment from multiple positions within the clinical interaction.

**Table 5:** Survivor and advocate-derived solutions to improve paramedic services with implementation needs required to enact those solutions identified by paramedics.

Suggested Solution	Paramedic Implementation Needs	Exemplary Quotes
Respect and Believe Survivors While Using Trauma- and Violence-Informed Approaches	Improve Organizational Culture Around IPV Deliver Expertly Designed Training and Education on: IPV Trauma- and Violence- Informed Practice Mitigating Bias and Discrimination Create Feedback Mechanisms for Performance Develop Mentorship Processes to Support Paramedics	“We need way more training because it can totally go wrong. Is this the right question? Is this the wrong moment? Lives are at risk, including our own. So having training would help structure that for us.” “[Emergency medical services] is sorely lacking in that hierarchy of power, and in that hierarchy of mentorship where you finish school, you get a license, and you sign onto a truck. And whatever happens next, it’s in your hands. And you have a lot of responsibility with none or not a lot of understanding as to what is acceptable, what is seen as professional, and what is seen as unprofessional.” “I think that we can build that trust. We can be their voice. We can be that safe person.” “I’ve developed a new respect for the soft skills in scenarios like that. And I will completely forego 90% of a medical evaluation to literally just sit there and hold someone’s hand and be a friendly face instead of a guy in a uniform providing a service. And then they feel better than if I did a 12 lead, a physical exam, like the whole thing. It doesn’t matter. It doesn’t matter if they feel like they’re just being poked and prodded with questions already by the police. They don’t need to be poked and prodded by me with medical questions. I can look at and evaluate you’re not that sick. So, we can just sit here for a minute. You can talk, I’ll listen.” “One of the things that we do in [emergency medical services], that gets paramedics engaged is when they see the positive outcomes…Seeing that positive feedback or getting some positive feedback is how you get paramedics to buy in. And then they study more, and they learn more about that that subject because they want to get that again...If they get really good at one thing, and they get positive results, and they see it. They do better at it, and they continue learning it. We don’t get any of that, generally, with victims of abuse.” “There is an actual want for this from paramedics…I think it’s well needed, and it will be well received. I think anything that comes out of this that helps us deal with these victims is good or fantastic.” “I think mentorship is important, and I think not having egos and bringing back mentorship is also very important. I think Fire excels in that. They excel in mentorship. They excel in public relations. Just watch with Nana, and they’ll go in, and Nana had some sort of bodily fluid on the floor for a period of time, but the first person to put their knee on the ground and say, “*how can I help*?” and hold her hand is Fire. The paramedics stand back, and don’t get dirty.”
Safe and Appropriate Direct Questioning About IPV	• Deliver Expertly Designed Training and Education to Paramedics on When, How, and What to Ask • Use, Make, or Adapt a Semi-Structured, Practical, and Adaptable Questioning Tool Aimed to Identify Reasonable Concerns • Be Conscious and Specific with Language • Have the Tool be Lenient – to Allow for Nuance – but Specific – to be Consistent	“Kind of like a [FAST-VAN; Face, Arms, Speech, Time, Vision, Aphagia, Neglect; widely accepted clinical tool] for violence where it’s this very specific, structured assessment.” “Yeah, that makes sense. So maybe then, having [an IPV screening tool] as like an assessment, that would be an option to fill out but still easy and streamlined, that would be good.” “The intention [of mandatory screening at hospital triage] was really good. The execution was quite poor. And so, this is kind of looking at a different way, where paramedics typically having one patient at a time, and a private space might have a good opportunity to ask those questions.” “I would never want to lose the personalness and the sensitivity of such questions to then just be like, you know, are your eyes open? Are your eyes closed? Do you answer me? Like there’s just so much nuance. I would worry that a form, or like some check boxes would take that personalization, the compassion, the empathy. It would get taken away by just checking some boxes.” “I think practice is a huge component of that, and recognizing it is okay to ask somebody, "*did somebody hurt you? Has this happened in the past? Are you concerned for your safety?*" I think those are very direct and very simple questions, to learn how to ask and to learn how to receive the answers too. I think the flaw in the system is that that it makes people nervous, and we see that in practitioners, we see that in our educators, and we see that in our instructors in school. It’s a topic that makes us nervous because we can’t do anything with that information right?”
Pathways to Care and Resources in Situations of IPV	• Create Specific Pathways for Survivors of IPV • Create Pathways for Situations of Conveyance and Non-Conveyance • Co-Develop Pathway Processes with Integration Into Other Services and Agencies • Create Specialty Teams Within Paramedicine for IPV-Caused Concerns	“So, ultimately, I think, actually dedicated like mental health or intake, just for dedicated resources would make a big difference for the transport side of things.” “It would be my ideal if I didn’t have to take every victim to a trauma centre for them to wait six hours to maybe talk to a nurse that might show them some compassion, give them the right resources, and point them in the right direction.” “I don’t see the reason why we can’t have [a process for survivors of IPV]. It frustrates me that we don’t. I think even police are going that way right now. They’re bringing in the [police and crisis team] in [redacted city] and stuff, and there’s more and more evidence showing that first responders need to be able to handle this vast array of victims and patients.” “We have special teams for so many different things in not just in [emergency medical services], but in in healthcare. You know, you have stroke teams, trauma teams, dialysis teams. They all come in and handle this, and once they get to the hospital there can be mental health specialists that come in. But I don’t see a reason why we can’t have resources in larger systems, at least in [emergency medical services] that we can call upon to attend on these calls and say, like, you know, I need the *this* unit.”
Referral	• Develop a Referral Process • Use Automation – Ideally Linked from Direct Questioning – to Initiate Referrals • Make the Referral Process Functional (e.g., simple, useable, quick, etc.) for Paramedics • Make the Referral Process Timely • Have Regional Considerations and Options • Use Common Language • Mobilize CPs in the Referral Process • Ensure Functionality (e.g., accessibility, utility, etc.) of Referred Resources for Survivors of IPV • Create a Referral Process for Situations of Conveyance and Non-Conveyance • Remove Barriers to Referral (e.g., referred resource follows up versus not left to survivor to reach out, etc.) • Provide Feedback to Paramedics Following Referral	“Yeah, it’s like when you go to the hospital and say we have a [GCS; Glasgow Coma Score; widely accepted clinical tool for assessing level of consciousness] of 8. That translates to everybody. Like you go in, say their [CIWA-AR; Clinical Institute Withdrawal Assessment Alcohol Scale Revised; widely accepted clinical tool for assessing threshold to treat for alcohol withdrawal] is 7 and they automatically know what they’re doing. So, we go in and say their domestic violence scale is 20 instead of 2 and then the person who actually needs to ask questions can ask them later when they’re feeling safe.” “You know I had a call a month ago, and the social worker actually called me. And now I did leave my number, because I was really trying to advocate for this family, and they called, and she just said, you know, “*thanks*”. You know, I don’t know what happened then, and it doesn’t really matter to me. But I did get that closure in the sense of that they’ve made a connection.” “Our community paramedic program is growing rapidly and they seem to be finding pitfalls where homecare doesn’t want to do this.” “I’m gonna feel better leaving that patient at home [with a referral] for my license, I’m gonna feel better about care provided to that patient. I’m gonna feel like there’s help when you walk away from those calls being like well, that was not a waste, because they need to be seen, but a waste, because nothing’s gonna happen like this sucks. And I’m gonna feel better about that [if the patients get a referral]. I would hope that would make patients feel better. I would certainly assume it does.” “If your domestic violence scale is a certain point score. And you click on it, that [patient care record] gets forwarded to somebody else who can look into it…The more automated it is, the better for us. I mean, we are so busy these days. That for us to make phone calls and refer or go through dispatch to call this person to call that person to follow up with them a week later. I don’t know. Like if there’s something on your [patient care record] that you go, domestic violence, they scored 15 and that’s enough for in two days the police make a welfare check to make sure this patient that has encountered domestic violence is still okay, it’s still a lot better.” “We have to check the silly little box in the [electronic patient care record] now, when it doesn’t even apply to our situation. It doesn’t apply to where we are in the province.” “Things that are maybe not explicit, but it’s suspected, and so in that kind of a process having something that paramedics can do where it advances the case to somebody that’s better positioned to provide those resources and those different pathways while saying as a paramedic, you don’t have to be 1,000% sure this is happening to potentially link someone to resources. Just that they’re going to have the option because of XYZ.”
Documentation into the Continuum of Care	• Create Guidelines for Paramedics on What, When, and How to Document in Patient Care Records • Build a Uniform and Trackable Section of the Patient Care Record for IPV Screening and Recognition • Integrate Selectable Fields for Consistency and Free Text Fields for Nuance • Ensure the Documentation Section is Simple, Easy to Use, and Mandatory • Soften Language (e.g., suspected IPV, possible IPV, requires investigation for potential IPV, etc.) to Mitigate Paramedic Definitiveness and Increase Uptake • Build Patient Care Record Software to Automatically Initiate Referrals in Cases of Known or Suspected IPV for Situations of Conveyance and Non-Conveyance, Reducing Paramedic Burden and Ensuring Continuity of Information Throughout the Process	“Yeah, knowing that my narrative doesn’t just go off into the cloud to never, ever be read. Yeah, that’d be great.” “What about just a simple click box that somehow connects, I mean this would be, I guess, in my little dream world. But literally it clicks “social work” or “social work consult” and then it flags somewhere further on. You know, like the social worker then is automatically looped in, or crisis response team, or whatever, right?” “I’m really trying to focus on who we’re painting a picture for. Any social worker, doctor that may read my story. Just so they really get a good understanding, an idea of what has happened in this patient situation. Just to kind of loop them in on everything I saw, everything that happened in front of me there, the patient’s own story, the police’s story, like, let me just kind of loop everything in and give them a good picture or good insight as to what they’re missing in the hospital.” “I agree with making it as easy as possible [to document and report], like maybe putting it somewhere, you can’t override it like the initial assessment. So, it’s just like triage that’s asking every single patient. That [IPV] just becomes one of our quick screens.” “[Paramedics] are in a bit of a different position to the rest of the healthcare service, in that we’re actually going into the home environment. So, we get to see that perspective. And it is our role to make sure we’re passing on that information.” “I think that this [IPV] patient would benefit from a consult with a social worker, or consult with whatever.” “I don’t see how you can incorporate the subtleties. However, I think something that then at the very least flags the potential for follow up with further services.” “Whether it ticks those boxes, and that’s what flags follow up with the services, so that it’s not down to the nuances of the individual paramedic to diagnose [IPV].”

### Paramedic-derived solutions to paramedic services challenges

For the seven most impactful challenges identified by the participants herein, participant-derived solutions, exemplary quotes, and action items can be found in Table 6. Solutions and action items had substantial overlap as the interrelated nature of the challenges yielded interrelated solutions and action items.

**Table 6:** Solutions, actions items, and exemplary quotes for challenges faced by paramedics when providing services to survivors of intimate partner violence (IPV).

Challenge	Solution	Exemplary Quotes	Action Items
Patient Barriers to Seeking Help	Reposition Paramedics as a Reliable Resource for Patients who are Experiencing IPV	“I find that it works well if you can just be quiet and give time and space. And, for us, sometimes they don’t want to talk and they’re not really saying much, and we have to allow them to have that platform to just share whatever they want when they want.” “We still need more training in regard to like de-escalation and violence in general.” “If you let everybody in the room know we’re just here to help. And kind of bring the tension down a little bit. Well, let’s take you over here and let’s take you over there. Let’s just talk.” “I mean, that can be hard, especially if that person fears that there might be something to hide. But I think that [the police] approach [separating the victim and perpetrator, creating two private spaces] works quite well when that person can now speak freely or even if we just, you know, go straight into the ambulance and just start going right there. It creates our own safe spot that we can work out of and get truthful answers out of.” “We do have a good ability to provide prehospital care and create a safe environment for these patients.”	• Have Paramedics Build a Safe Environment for Patients to Enhance Approachability • Separate the Patient and Perpetrator, Being Conscious of Scene Dynamics, to Enable the Ability to Respond Openly • Ensure that Patients can Consistently Rely on Paramedics as a Useful Resources to Deliver Support • Be Conscious of the Gender of Responders and, Where Possible and Safe, Access a Paramedic with the Same Gender as the Patient • Be Conscious of Inherent Power Dynamics Between Uniformed Personnel and the Public • Equip Paramedics with De-Escalation Training to Reduce Tension and Effectively Provide Support
Individual Paramedic Disposition	Develop a Culture of Psychological Safety for Practitioners and Patients, and Facilitate Sustainable and Reasonable Working Conditions for Paramedics	“The other one that came to mind is we are all human as paramedics. We’re all human and, you know, sometimes we don’t actually have capacity to give to these people with these mental health challenges or, you know, whatnot because we’ve just been run ragged with 10 calls of nonsense.” “We have colleagues going to Fire that look great. They look 10 years younger. They’re eating healthy meals. They can sign up for a fitness trainer. They have access to psychologists and just more of a team atmosphere whenever they want. So, I think just again going back to the basics is that paramedics need to be able to take better care of themselves, to be able to connect, and be there for each other again.” “There’s no way that you can expect some 19-year-old paramedic, with no life experience, who’s just finished school to come out and be good at this. And they’re absolutely not the people that I would want talking to my loved ones if they were victims of any form of violence, really or frankly, for any emergencies. I think if we were to add that mentorship, that it would go a long way as well.” “I think just more awareness, more cultural training, and more sensitivity training.” “Maybe we just walked out of cardiac arrest. And now we walk into a home of violence and, so, I just think more normalizing the ability to say, I need five minutes after this call rather than clearing and right away just go. So, that when we actually walk into these homes, we have capacity to be present for our patients as we would all like to be.”	• Build and Maintain a Culture of Staff Well-Being and Psychological Safety within Paramedic Services • Create Mentorship Programs within Paramedic Services to Continue to Train and Support Safe and Equitable Paramedic Practice • Improve the Team Atmosphere of Paramedic Services • Improve Incentives to Practice as a Frontline Paramedic • Pay Paramedics to Build and Maintain Their Personal Health and Fitness • Facilitate Easy Access to Mental and Physical Health Supports for Paramedics • Effectively Use Paramedic Resources for Appropriate Calls • Limit Paramedic Resource Utilization to Avoid Excessive Workloads and Burnout • Remove Counterproductive Middle Management from Paramedic Services • Improve Hiring Practices to Acquire High Calibre Staff • Establish Maintainable Career Longevity Infrastructure for Retainment of High Calibre Employees
Individual Paramedic Confidence	Build Self-Efficacy in Practitioners, and Develop Practical Care Options so Paramedics Have the Means to Meaningfully Support Survivors of IPV	“I like that there’s this discussion being had right now and am hopeful to see some more training, especially with simulations and some practice in approaching these delicate subjects. I know a lot of people are very uncomfortable asking the direct questions to people, that they have suspicions of either being a perpetrator or being a victim.” “You’re going to an untrusted environment to begin with, probably a history of bad experiences at the hospital. So, how do we have tools that are going to build trust.”	• Use Targeted Education and Training to Build Self-Efficacy (e.g., mastery, verbal persuasion, modelling) Among Paramedics • Develop and Provide Paramedics with Tools to Build Trust • Build Systemic Infrastructure (e.g., point-of-care tools, patient pathways, etc.) for Paramedics to Use to Support Survivors of IPV • Show Paramedics the Results of Their Actions to Reinforce the Behaviour • Enable Simple Practices to Have a Larger Impact (e.g., screening for IPV that automatically refers eligble patients, etc.)
Paramedic Service Education, Training, and Readiness	Appropriately Educate Paramedics with Functional Information, Train Paramedics with Practical Skills and Behaviours, and Build the Readiness (targeting the constructs of readiness) to Encounter Patients Experiencing IPV	“Since we’ve had this [IPV] patient, we’ve actually ended up having a training session with their [victim services] coordinator. She came in and gave us quite a bit of information about how we can help the victims a lot more effectively and what resources are actually available to us here in our local community as well as regionally.” “I really like the concept of the [community paramedics] being specially trained to recognize and respond to intimate violence calls and bridge that gap between [emergency medical services] and social work.” “I wish that that had been standard, and that information had come across a lot earlier cause there was a little bit more that could have been done, or maybe it could have been done a little bit earlier. More chances.”	**Design and Delivery** • Broaden the Scope of Paramedic Education and Training to Encompass the Realities of the Role • Co-Design and Develop Education and Training with Field Experts • Deliver Education and Training through Various Modalities to Accommodate Learning Preferences • Have Qualified Personnel Deliver Education and Training (i.e., not paramedics on accommodations, credible educators and instructors, etc.) • Make Recurring IPV Education and Training Mandatory **Content** • Educate Paramedics: ◦ on IPV ◦ on Social Determinants of Health in Relation to IPV ◦ on IPV-Caused Brain Injury and Non-Fatal Strangulation ◦ on Safety and Lethality in Situations of IPV • Train Paramedics: ◦ on De-Escalation Techniques and How to Manage On Scene Dynamics ◦ to Deliver Trauma- and Violence-Informed Practice ◦ to Create Safe Spaces and Time for Survivors of IPV ◦ to Screen for IPV ◦ to Recognize Signs of IPV ◦ to Respond Appropriately to Disclosures of IPV
			◦ on Standards of Practice for Cases Involving IPV ◦ to Recognize Unsafe Situations, Know When to Involve Police, and Build Basic Safety Plans ◦ on Documentation Standards and Processes ◦ to Utilize Practical Pathways and Processes to Meet Patient Needs through Resources and Referrals ◦ to Preserve Patient Autonomy ◦ to Transport Patients to Destinations that Meet Patient Needs ◦ to Be Able to Manage Situational Logistics (e.g., children on scene, transport concerns, etc.) ◦ to Properly Debrief and Practice Self-Care
Paramedic Service Guidance	Establish and Maintain Evidence-Based Clinical Practice Guidance for Paramedics in Situations Involving IPV	“Yeah, it’d be nice to have specific questions for situations like that. Like a focused assessment. Like we have specific assessments for heart attacks, we have specific assessments for neuro, why can’t we have a specific assessment for violence?” “If we could do like a huge training, and maybe that becomes every paramedic in the province with the training.” “I do think we’re an emergency service and we need to deal with the sensitivities of a huge variety of situations.” “Since we are oftentimes the first medical contact for these patients, for these victims, it could be, it should be broadened so we have an accurate understanding of what resources are available and what options are there.”	• Broaden the Scope of Clinical Practice Guidance within Paramedicine to Meet the Realities of the Profession • Develop Guidance Around Scene Management • Generate Evidence-Based Clinical Practice Guidance for IPV, Specifically: ◦ Paramedic-Specific Screening for and Recognizing Signs and Symptoms of IPV ◦ Responding Appropriately to Disclosures of IPV ◦ Activating Paramedic-Initiated Pathways and Processes to Resources and Referrals ◦ Documenting in a Correct Manner that Supports all Facets of Patient Care • Develop Guidance for Safety and Lethality Assessment and Processes When Identified • Develop the Role and Scope of Community Paramedicine Programs and Specialized Teams to Create In-Service Expert Resources • Develop Guidance Around In-Service (e.g., CP teams, specialized paramedic teams, etc.) and Out-of-Service (e.g., police and crisis teams, crisis response teams, etc.) Referral Teams • Develop Functional Guidance for Logistical Considerations (e.g., children, transport, etc.) • Develop Guidance Around Transport Destination Options that Best Meet Patient Needs • Develop Guidance on Facilitating Transitions along the Continuum of Care
Paramedic Service Configuration	Develop and Maintain Systemic Infrastructure to Create the Ability for Paramedics to Adhere to Clinical Practice Guidance	“[Interacting with paramedics] gives them a second opportunity. And maybe that’s the time that they’re talking to the perpetrator and it’s bad, and they go “*now I understand that you’re right. I don’t have to live like this*.” Like, that amount of time can just change the world.” “I think [community paramedics] really have a huge ability to play role in mental health stuff and make a difference with like sustained contacts and all that kind of thing.“ “The [community paramedic] role could thrive on bridging emergency services and social work.” “It would be nice if we had specialty teams of paramedics that could be specifically requested to come in, and at least they could do two things; one, they’re bringing some form of expertise, as well as the [emergency medical services] care required to treat wounds and injuries and two, identify life threats simultaneously. I don’t see a reason why we can’t have that. It frustrates me that we don’t. I think even police are going that way.”	• Create and Maintain Policy and Procedural Guidance to Facilitate All Service Configurations • Create and Maintain a Flagging System and Data Base for Known Addresses or Patients with IPV Considerations • Create and Maintain Patient Care Record Infrastructure for IPV, Specifically: ◦ Trackable Fields for Monitoring IPV ◦ Mandatory, Simple, Brief, and Functional Paramedic-Specific IPV Screening Tool Software (selectable field and free text field options) ◦ Automate Screening Tool Software to Initiate Referrals for Known or Suspected IPV ◦ Ensure Prompt Feedback Provided to Paramedics in Cases of Referral Initiation ◦ Safety and Lethality Assessments and Processes When Identified • Create and Maintain Processes and Pathways to Care for Survivors of IPV, Specifically: ◦ for Situations of Conveyance ◦ for Situations of Non-Conveyance ◦ with in-Hospital Referrals ◦ with in-Community Referrals ◦ Processes that Integrate Inter-Agency Communication ◦ Processes that Contribute to a Data Base that Tracks Patient Progress and Situates them Appropriately ◦ Processes that Meaningfully Support Survivors of IPV • Expand and Maintain the Role and Scope of Community Paramedicine Programs and Specialized Teams to Create In-Service Expert Resources • Create and Maintain In-Service (e.g., CP teams, specialized paramedic teams, etc.) and Out-of-Service (e.g., police and crisis teams, crisis response teams, etc.) Referral Teams • Create and Maintain Options for Logistical Considerations (e.g., children, transport, etc.) • Create and Maintain Transport Destination Options that Best Meet Patient Needs • Create and Maintain Processes Facilitating Transitions along the Continuum of Care
Interagency Networks	Join Forces with Other Agencies that also Serve Survivors of IPV to Generate a Collaborative and Communicative Network of Overlapping Resources to Better Meet the Needs of Survivors of IPV	“If everyone’s talking and everyone’s kind of collaborating on this and trying to come towards a solution together, [it will work better].” ‘Let’s get proper, like a hundred percent, continuity of care.” “Then [the patient] would talk to someone, a real person, and have actual resources, that will give them access to all the resources that they need to get this person into a safe situation.”	• Build and Leverage Relationships with Relevant in-Hospital and in-Community Services • Establish a Process with Outside Service Providers to Holistically Meet Patient Needs via the Overlapping Network of Services • Mobilize Resources Outside of Paramedicine, when Appropriate, to Reduce Call Volumes and Paramedic Burden • Allow Paramedics to Initiate Referrals to Other Services in-Community • Form Integrated Teams with Providers from Other Services • Ensure Continuity of Safety into the Emergency Department and Other Services • Facilitate the Transfer of Information to Appropriate Services to Permit Service-Initiated Follow Up and Referral for Survivors of IPV • Share Information (e.g., documentation, etc.) Among Services to Ensure All Services are Aware of Ongoing Situations • Allow Paramedicine to be the Vital Link from Patient to Services, Including Services External to Paramedicine

## Discussion

This study examined how the perspectives and experiences of paramedics may inform our understanding of current clinical practice and guide potential improvements for paramedicine. The results herein are the first to learn from the valuable perspectives of paramedics about the challenges and possible solutions at the intersection of paramedicine and IPV. This work highlights perspectives outlining the current professional environment described by a sample of paramedics working in Canada, creating tangible experience-based evidence of generally adverse working conditions but with dichotomous potential hope for improvement. This research expands on a growing body of literature outlining, from the paramedic perspective, challenges faced at the intersection of paramedicine and IPV. Promisingly, solutions and action items to move past or mitigate these challenges were also identified by paramedics. The perspectives and experiences of paramedics about the intersection of paramedicine and IPV add depth and structure to the existing body of research, aiming to progress the field and stimulate positive change.

Context is paramount. The perspectives of paramedics about working in paramedicine, irrespective of IPV, provide a contemporary appraisal of the working environment. Non-paramedics may be unfamiliar with the daily circumstances faced by the contemporary paramedic. While many challenges have been alluded to in paramedicine [31–33], the raw and current perspectives discussed in this research provide a layer of context that is otherwise absent in recent years. Furthermore and in the context of paramedicine and IPV, considerations and nuances from other applied disciplines and environments, like law enforcement and non-paramedic HCPs (e.g., in-hospital, in-clinic, etc.), may be useful starting blocks to guide paramedic-specific solutions and action items but are unlikely to be directly transferrable. Many practices have not transcribed well directly from medicine to paramedicine, and overlooking the specific discipline and environment can attribute to these translational shortcomings.

The marked frustrations reported by participants indicate a discrepancy between what paramedics wish to be able to do to support and provide care for survivors of IPV and what they are currently doing. This provides evidence indicating a need exists at the intersection of paramedicine and IPV. These findings are in alignment with survey research from a sample of paramedics, reporting that paramedic readiness to encounter patients experiencing IPV has insufficiencies [20]. Further, the identification of this need has been supported in research among survivors of IPV and IPV advocates [23], respectively.

Although paramedics are distinctly unique in their intersecting role between emergency response and healthcare, similarities exist when considering the challenges faced by both law enforcement personnel and HCPs. Among law enforcement personnel, inappropriate attitudes, inaction, and incomplete service provision were shared challenges with paramedics [34,35]. Similarly, lack of recognition, lack of training, fragmented infrastructure, feeling ill prepared, and lacking readiness were consistent experiences among HCPs and paramedics [36–38]. While shared experiences among service providers can highlight commonalties, the context and environment for each responder group should be considered and generalizations among service providers should be made extremely cautiously.

The challenges reported by paramedics at the intersection of paramedicine and IPV were plentiful. Challenges spanned patient, individual paramedic, paramedic service, and systemic domains. Complexities exist when considering that even if certain challenges can be mitigated, others may still impair the progression of survivors to appropriate care and resources. All links in the progression of survivors to appropriate care and resources must be intact and functional to achieve the highest likelihood of success.

A lack of absolute certainty of abuse or neglect was previously documented as a barrier to action by paramedics [39]. This was mirrored in the findings here in and indicates this issue has yet to be meaningfully addressed almost 30 years on. Additionally, participants identified the role of paramedic gender within these interactions, primarily highlighting paramedics who identify as women may have more success engaging with survivors of IPV who identify as women. Previous ideas have circulated about having paramedics who identify as women respond to calls involving IPV or sexual assault, but this notion can disproportionately burden paramedics who identify as women with trauma exposure. Further investigation is warranted to balance patient comfort and paramedic safety.

The solutions identified by survivors and advocates were also identified by paramedics prior to those discussions. The alignment between the three populations indicates a cohesive direction to advance the field. The cohesive solutions and action items from this research represent the best evidence-based direction to advance paramedicine at the intersection of IPV. The existing landmark guidance for paramedicine in Australia advocating for paramedics to recognize, respond, refer, and record IPV is also in alignment with the findings of this research [40]. This indicates the Australian guidance may be translational and adaptable to other geographic regions and contexts. Meaningful implementation of the action items to target the solutions that address the problems should be pursued.

Helping paramedics and paramedic services to see IPV as a genuine concern is not the primary challenge faced by this research. The concept that IPV is an unacceptable circumstance that a large portion of patients endure, and that paramedics are uniquely situated to intervene when encountering people experiencing this circumstance is generally accepted [20,40]. The challenge lays in what comes next. The development of education and training without the clinical practice guidance and systemic infrastructure to deploy the new concepts and use the learned skills is unlikely to be effective. Just as the development of clinical practice guidance and systemic infrastructure without the development of supporting policy, procedures, and regulation is likely to be ineffective. The most effective approach based on the available evidence indicates that simultaneous development of education and training, clinical guidance and systemic infrastructure, and policy, procedures and regulation is likely to facilitate change. Although this can appear daunting, it can be compartmentalized into manageable tasks if done in a strategic manner.

While detailed implementation research design is outside of the scope of this project, considerations for mobilization of evidence-based implementations remain essential in advancing paramedicine. The Knowledge-to-Action framework is well established in other fields as a logical guide to address knowledge-practice gaps [41,42]. The Knowledge-to-Action framework has been applied to paramedic system modernization [43], highlighting utility within the discipline. Using established implementation theories, models, and frameworks may increase the likelihood of successful implementations. Knowledge mobilization is an area of great opportunity within paramedicine. Using effective strategies can save time, money, and anguish.

### Limitations

The participants of this study represent valuable opinions and perspectives. However, in relation to the gross number of paramedics in Alberta, British Columbia, and Saskatchewan, the findings herein represent the participants and extrapolation to all paramedics may not be appropriate. Three provinces out of the 13 Canadian provinces and territories were represented in this study. Although universal similarities exist among paramedics, regional and contextual considerations should be applied when interpreting this data. The ID approach imposes the risk that interpretations of the data source may not reflect the raw perspectives of participants. Future research should consider gathering the perspectives of patients and paramedics in additional regions and contexts of interest. Professional demographic data (e.g., years in paramedicine, year of entry-to-practice training) was not collected, limiting the ability to describe the participants. The personal experiences with IPV outside of paramedicine may have impacted the input from participants. Conducting focus groups may have imposed on the acceptability of responses in this group setting. The focus group setting can also impose power and social dynamics. Variation in focus group size and modality may have impacted group dynamics and information-sharing. Front-line paramedics may not fully understand organizational or systemic implementation needs. Thus, future research should investigate the perceptions and experiences of leaders in paramedicine.

## Conclusion

This research adds to the growing body of literature at the intersection of paramedicine and IPV. This research provides clear evidence from those on the frontlines who have recognized gaps in service delivery. The experiences and perspectives of the paramedics who participated in this study outlined challenges at the patient, practitioner, organizational, and systemic levels. Promisingly, solutions and action items to guide implementations to address these challenges were generated. While each individual attempt to progress the field requires contextual considerations, the concepts from this research contribute to informing evidence-based practice for paramedics and paramedic services. The findings from this research contribute to the existing body evidence and further substantiate the call to action that prompts paramedic services to embrace this opportunity and mobilize the existing evidence towards for positive, evidence-based change. Ambulance calls for IPV are happening every day, and it is time paramedicine engages with teachings from this growing body of literature to answer appropriately.
